# Current Perspective on MDMA-Assisted Psychotherapy for Posttraumatic Stress Disorder

**DOI:** 10.1007/s10879-017-9379-2

**Published:** 2018-01-06

**Authors:** Sascha B. Thal, Miriam J. J. Lommen

**Affiliations:** Department of Clinical Psychology and Experimental Psychopathology, University of Groningen, Grote Kruisstraat 2/1, 9712 TS Groningen, The Netherlands

**Keywords:** PTSD, MDMA, MDMA-assisted therapy, Substance-assisted therapy, Overview

## Abstract

The present paper discusses the current literature with regard to substance-assisted psychotherapy with Methylenedioxymethamphetamine (MDMA) for posttraumatic stress disorder (PTSD). The aim of the paper is to give a comprehensive overview of the development from MDMA’s early application in psychotherapy to its present and future role in the treatment of PTSD. It is further attempted to increase the attention for MDMA’s therapeutic potential by providing a thorough depiction of the scientific evidence regarding its theorized mechanism of action and potential harms of its application in the clinical setting (e.g., misattribution of therapeutic gains to medication instead of psychological changes). Empirical support for the use of MDMA-assisted psychotherapy, including the randomized, double-blind, placebo-controlled trails that have been conducted since 2008, is discussed. Thus far, an overall remission rate of 66.2% and low rates of adverse effects have been found in the six phase two trials conducted in clinical settings with 105 blinded subjects with chronic PTSD. The results seem to support MDMA’s safe and effective use as an adjunct to psychotherapy. Even though preliminary studies may look promising, more studies of its application in a psychotherapeutic context are needed in order to establish MDMA as a potential adjunct to therapy.

## Introduction

Posttraumatic stress disorder (PTSD) is a trauma- and stress-related disorder with close links to anxiety-, and dissociative-disorders. It is caused by exposure to a traumatic event that could result in serious injury or the loss of one’s life. Events that can be considered traumatic may further include being witness to or coming to know of close relatives or friends being affected by trauma events as well as being repeatedly confronted with aversive details of a traumatic incident. As a consequence, extreme psychological distress, recurring dreams of traumatic events and flashbacks are common symptoms experienced by patients suffering from PTSD (American Psychiatric Association 2013). Traumatic reminders may trigger prolonged and intense distress as well as physiological reactivity. Thus, external reminders and thoughts or feelings related to the trauma are often avoided. Further, negative changes in mood and cognition, and alterations in arousal and reactivity may be observed (American Psychiatric Association 2013). Lifetime prevalence of traumatic life events is estimated to revolve around 50–90% globally (Wittchen et al. 2009). Nevertheless, the estimated lifetime risk for PTSD reported by Kessler et al. (2005) revolves around 6–10%. Kilpatrick et al. (2013) estimated lifetime prevalence to amount to 8.3% in the United States using DSM-V criteria, with women being twice as likely to develop PTSD compared to men (Wittchen et al. 2009).

There is a variety of treatment options available, with psychotherapy being recognized as the most effective form of treatment for PTSD (Van Etten and Taylor 1998). Trauma-focused therapies like Cognitive Behavior Therapy (CBT), Cognitive Processing Therapy (CPT), Trauma-Focused Cognitive-Behavioral Therapy (TFCBT), and Eye Movement Desensitization and Reprocessing (EMDR) constitute widely suggested first line treatments for PTSD (Benedeck et al. 2009; Cloitre 2009). In a meta-analysis, Bradley et al. (2005) found different trauma-focused therapies to display equal efficacy for PTSD patients, with clinical improvement being evident in 44% of those entering treatment. In contrast, treatments that do not distinctly address processing of traumatic contents were found to be less effective in the reduction of PTSD symptoms (Flatten et al. 2011). A problematic issue psychotherapy has been dealing with is the high drop-out rates of around 30% (Cloitre 2009). These high dropout rates may be explained by the detrimental effects trauma exhibits on the patients’ ability to form trusting interpersonal relationships, subsequently affecting the working alliance between the patient and therapist (Doukas et al. 2014). Additionally, therapeutic effectiveness may be limited by the short period of optimal arousal (i.e., therapeutic threshold) displayed by many individuals with PTSD, further contributing to higher dropout rates. Interferences in autonomic arousal may restrain the process of reforming fear structures targeted by the psychotherapeutic treatment (Foa and Kozak 1986). These issues may explain findings showing that remission of symptoms after 40 months is only achieved in around 44% of those undergoing treatment (Morina et al. 2014).

Of those diagnosed with PTSD, about 20–30% respond to pharmacotherapy (Stein et al. 2009) with sertraline and paroxetine (Jeffreys 2009). Selective serotonin reuptake inhibitors (SSRIs) were, as a class, found to have small effect sizes in PTSD symptom reduction (Hoskins et al. 2015) and are thus recommended as second-line treatment options (World Health Organization 2013). The use of benzodiazepines was found to negatively impact treatment outcomes in PTSD (Van Minnen et al. 2002). The need for future research into more effective agents for the treatment of PTSD was emphasized by several reviews of PTSD treatment studies (Foa et al. 2009; Stein et al. 2009). Currently existing treatment methods are ineffective for 25–50% of patients enrolled in clinical trials (Foa et al. 2009; Stein et al. 2009; Mithoefer et al. 2011). As reported by the Guardian, more US soldiers died in 2012 by committing suicide than being killed in combat (Pilkington 2013) and the economic costs of PTSD and trauma- and stressor-related disorders are estimated to amount to 43.2 billion dollars annually (Greenberg et al. 1999). The necessity for more effective treatments efficiently reducing current treatment failure rates thus becomes apparent. A recently reintroduced psychopharmacological adjunct to psychotherapy yields promising results: 3,4-Methylenedioxymethamphetamine (MMDA).

## What is MDMA?

MDMA is a ring-substituted amphetamine with structural similarities to mescaline (Green et al. 2003). Even though traditionally regarded a psychedelic amphetamine, considerations have been made to classify MDMA as entactogen—establishing a unique class of drugs (Nichols and Oberlender 1990). It was first synthesized in 1912 and patented in 1914 (Benzenhöfer and Passie 2006), but its psychoactive properties in humans were not studied until 1978. In 1985 MDMA was emergency scheduled and categorized as a Schedule I drug by the Drug Enforcement Administration (DEA) causing severe restriction of all clinical research. Subsequent illicit use, however, continued and increased (Sessa and Nutt 2007).

Positive cognitive effects of MDMA in a controlled clinical setting were described to include enhanced mood and well-being, happiness, relaxation (physical and mental), increased emotional sensitivity and responsiveness, heightened openness, extroversion and sociability, the feeling of closeness to other people, slight (visual, auditory, and tactile) changes in perception and exceptional anxiolysis (Harris et al. 2002; Vollenweider et al. 1998, 2002, 2005). Anxiolytic effects of MDMA comprise the feelings of immediate threat through the creation of a sense of detachment in patients rather than only diluting the very feeling of anxiety. Theoretically, this would allow for analytic reflection of past situations from different emotional perspectives (Schuldt 2015). The effects of MDMA usually peak 2 h after admission and with elimination half-life revolving around 8 h (Cole and Sumnall 2003; Mithoefer et al. 2011), it coincides with average durations of substance-assisted treatment sessions (Mithoefer 2016). Most importantly, aforementioned pharmacological and subjective effects of MDMA have been consistently established across clinical settings (Kirkpatrick et al. 2014). For a more thorough analysis of positive and negative effects as well as the physiological effects following MDMA induction in clinical settings, please resort to Schuldt (2015).

## Why May MDMA be a Suitable Adjunct to Trauma Therapy?

Since the first paper on MDMA’s effects on humans was published in 1978 (Shulgin and Nichols 1978) and its prohibition in the United States in 1985, MDMA was amply applied as a catalyst in the psychotherapeutic process (Grinspoon and Bakalar 1986). Nevertheless, there appeared no systematic scientific literature on the early use of MDMA in clinical settings until the mid-1980s (Greer and Tolbert 1986). Research regarding its potential for psychotherapy came to a halt after its illegalization. Only during the last decade therapeutic trials with subjects with psychiatric diagnoses have been re-approved and conducted again (see Bouso et al. 2008; Mithoefer et al. 2011; Oehen et al. 2013).

Approaching the current rationale for the application of MDMA in therapy from a psychotherapeutic perspective, its efficacy is hypothesized to result from its positive psychological effects. Positive outcomes in PTSD therapy show a firm relationship with the strength of the therapeutic alliance (Charuvastra and Cloitre 2008), simultaneously, forming beneficial interpersonal relationships based on trust is often difficult for PTSD patients (Doukas et al. 2014). A small window of optimal arousal or therapeutic threshold, frequently producing distress and at times dissociation, is observed in many PTSD patients (Foa and Kozak 1986). Some of these challenges might be attenuated by MDMA, as the pharmacological effects of MDMA include an increase in the neurohormones oxytocin, prolactin and cortisol and in the monoamine neurotransmitter serotonin (Grob et al. 1996; Harris et al. 2002; Wolff et al. 2006; Nichols et al. 1982). Oxytocin is suggested to play a role in the accurate perception of emotion, affiliation and trust (Kirsch et al. 2005; Zak et al. 2005), highlighting its potential value in assisting with the formation of a therapeutic alliance. Thus, it might help to revisit traumatic experiences in a state of emotional engagement (Mithoefer et al. 2011). Elevated levels of oxytocin may improve social support and bonding (Olff 2012; Frijling et al. 2014), increase trust (Baumgartner et al. 2008), and decrease amygdala activation (Kirsch et al. 2005) outside the therapeutic context in those suffering from PTSD. Increases in emotional empathy and prosocial behavior (Hysek et al. 2012) as well as a positive bias to socio-emotional stimuli (Kirkpatrick et al. 2014; Bedi et al. 2009) may also foster therapeutic progress. Since threatening interpretations are reinforced by negative emotional states (Mathews 2006), elevated levels of serotonin after MDMA ingestion may diminish this effect by increasing self-confidence and reducing feelings of anxiety and depression. Hence, this might help people to approach past experiences from different perspectives (Sessa 2011). According to the Emotional Processing Theory (EPT) of exposure therapy (Foa and Kozak 1986), fear reduction is achieved when information incompatible with the fear structure is incorporated. To do so, attending to threat is essential. MDMA may facilitate this very process. Negative cognitive effects in controlled clinical settings were observed to include disturbances of thought and difficulty concentrating, accelerated thinking, thought blocking, and impaired decision making (Vollenweider et al. 2002). In summary, these findings point to MDMA as a valuable catalyst disburdening cooperative engagement in therapy by strengthening the therapeutic alliance and by enhancing the identification of and response to emotional states.

Revisiting the trauma is often associated with intolerable negative feelings making confrontation extremely difficult. PTSD patients exhibit an attentional bias towards threat related stimuli, which correlates with amygdala activity. They also display increased reactivity to these stimuli (El Khoury-Malhame et al. 2011). During a MDMA-assisted therapy session traumatic memories are, however, often experienced as less threatening and may thus facilitate reconsolidation of threatening memories (Doblin 2002). This may be explained by the positive correlation between decreased blood flow in the right amygdala and right hippocampus and the intensity of subjective effects of the experience (Carhart-Harris et al. 2015) and the observation that favorable autobiographical memories are perceived as more vivid and intense, while unfavorable memories are regarded as less negative and less distressing (Carhart-Harris et al. 2014). Bremner et al. (2005) found strong left amygdala activation, in participants with PTSD who underwent a conditioned fear paradigm, while the anterior cingulate cortex appeared deactivated. In comparison, decreased amygdala activation and increased anterior cortex activation was established in participants while reporting their worst memories after MDMA ingestion (Carhart-Harris et al. 2014). Again, it may be reasoned that the effects of MDMA on aforementioned brain areas foster memory reconsolidation. By what Amoroso (2015) describes as ‘mirror image of neural activation’, displayed in PTSD patients faced with fear after taking MDMA compared to those who confronted fear without having taken MDMA beforehand, imaging studies visualize possible changes in memory perception and perspective (for a detailed depiction of these changes see Sessa 2011). In addition, Gamma et al. (2000) evidenced increased cerebral blood flow in the occipital and ventromedial frontal cortex following MDMA administration. The neurocircuitry model of PTSD assumes absence of extinction of fear to be mediated by the ventral/media prefrontal cortex and the amygdala (Rauch et al. 2006). Besides, raised cortisol levels were found to enhance the extinction of fear in psychotherapy (Dominique et al. 2011). Taken together, the effects mentioned above might prevent the patient from feeling overwhelmed while confronting the trauma. Patients may still be able to access the memory of the traumatic event, even though feeling detached from the sense of imminent threat, which may facilitate reconsolidation of these memories.

Heightened amygdala activity is also associated with fearful and threat-related social stimuli (Whalen et al. 2001), and relatedly a study by Bedi et al. (2009) evidenced MDMA to reduce left amygdala activity in response to angry facial expressions. These findings may help to illuminate MDMA’s positive effects on social behavior and anxiety. Also, heightened activity in the ventral striatum, which is important for the processing of (socially) rewarding stimuli (Haber 2011), after attending to happy facial expressions was assessed in the same study. Collectively, these findings may provide further evidence for MDMA’s potential to improve the therapeutic relationship (Schuldt 2015). Ultimately, current evidence suggest that combined psychological, prosocial and anxiolytic effects of MDMA may contribute to trauma therapy.

## Potential Harmful Effects Associated with the Application of MDMA in the Therapeutic Setting

The safety of MDMA is a rather controversial topic in the scientific literature as well as in the general public. In the therapeutic context acute adverse side effects may encompass nausea, vomiting, jaw clenching, muscle aches, feelings of numbness, headache, dizziness, fatigue, sweating and decreased appetite (Baylen and Rosenberg 2006). Detrimental effects on neurocognition include deficits in higher cognition and retrospective memory, depressive and confused thoughts, disturbed sleep and reduction of serotonin transporter levels in the cerebral cortex, amongst others (for an extensive review and debate see Parrott 2013; Doblin et al. 2014; Multidisciplinary Association for Psychedelic Studies 2016). Nevertheless, the Food and Drug Administration (FDA) assessed the benefit risk ratio to be acceptable for clinical studies of MDMA-assisted psychotherapy (Doblin 2002). Arguably aforementioned harmful effects may not be generalizable to MDMA-assisted psychotherapy for several reasons.

Firstly, MDMA and “Ecstasy” have been used interchangeably in the past. Whereas the former depicts the abbreviated version of a single chemical compound, references to “Ecstasy” often correspond to tablets containing MDMA alongside other components, e.g., 4-methylmethcathinone (mephedrone; Brunt et al. 2011), raising impurity issues (Spruit 2001). In order to appropriately assess the dangers of MDMA in the therapeutic context studies exploring the effect of high-quality product (*Good Manufacturing Practice*, or GMP-MDMA), imperatively used in current trials, may be of higher validity. Additionally, it is important to highlight that environmental conditions commonly modulate the psychobiological effects of MDMA (Parrott 2004, 2006) further questioning the comparability of recreational and therapeutic use. It is thus debatable whether therapeutic doses of GMP-MDMA constitute significant risks for long term harmful effects, when administered in controlled environments (Vollenweider et al. 1999, 2001). The controlled clinical trials conducted up to date report no persisting drug-related harm in over 850 participants (Doblin et al. 2014; Mithoefer et al. 2011, 2013). Therefore, the interchangeable use of the terms MDMA and ‘Ecstasy’ should be avoided in scientific literature regarding MDMA-assisted psychotherapy and more studies assessing long-term harmful effects of GMP-MDMA are needed.

Secondly, as coherently depicted by Amoroso (2015), most of the studies concerning the effects of MDMA on humans may not be generalizable to its application in therapy. Whereas a study by Schilt et al. (2008) investigated cognitive deficits in participants averaging a lifetime exposure of 327 tablets, it was reported that no more than 20–30% of ecstasy users consumed more than 25 tablets in their entire lives (De Win et al. 2005). Moderate and heavy ecstasy-users only differ slightly on a battery of neurological test, but more so on measures of impulsivity and mental processing, possibly contributing to poly-drug use and neurocognitive deficits (Halpern et al. 2004). In addition, causation may not imperatively be implied by correlation (Aldrich 1995). It is generally complicated to control for poly-drug use, product purity and dose and underlying as well as preexisting mental disorders (Amoroso 2015). The possible involvement of confounding variables admonishes scientists to interpret those findings with caution (Lieb et al. 2002). Again, the extent to which such findings can be transferred to MDMA-assisted therapy remains questionable.

Thirdly, some of the misconceptions about MDMA may be due to flawed studies. A paper, later retracted from *Science*, claimed MDMA to cause severe dopaminergic neurotoxicity and subsequent death in primates (Ricautre et al. 2002). Advertising campaigns portraying “Ecstasy” as a compound generating holes in brain tissue were supported by aforementioned government funded research (Schuldt 2015). Upon critical responses questioning the validity of those findings (Mithoefer et al. 2003), it was found that the use of methamphetamine instead of MDMA caused the experimenters to obtain neurotoxic results.

Fourthly, MDMA’s adverse effects include tolerance and withdrawal symptoms (Degenhardt et al. 2010), but it does not seem to be linked to more serious adverse effects like suicidal tendencies that have been associated with paroxetine (Le Noury et al. 2015), which is currently used in the treatment of PTSD. Furthermore, the evidence for the dependence potential of MDMA seems limited. It should be kept in mind that these findings often refer to recreational use and may thus not be generalizable to studies investigating its therapeutic potential.

A potential concern in the use of MDMA as an adjunct to psychotherapy is the risk of patients misattributing therapeutic gains to medications minimizing the maintenance of improvements through psychological changes. As a consequence, the patients may be at higher risk of relapse, have more severe withdrawal symptoms and a greater loss of gains (Başoğlu et al. 1994). Preliminary evidence from follow-up studies to clinical trials with MDMA-assisted psychotherapy, however, indicates that clinical and statistical increases in symptom relief may persist over time (Mithoefer et al. 2013; Oehen et al. 2013). In this regards, MDMA use in therapy might be different from other pharmacological interventions that are prescribed on a daily basis. Nevertheless, the risk of misattribution and subsequent reduction of positive effects acquired through psychological changes deserves further investigation and should be considered for discussion with patients.

Eventually, these issues do not support inconsiderate use of MDMA or claim its use to be hazard-free, but rather underline the immediate need for more research directly examining its safe usage in the therapeutic context. Abuse-oriented paradigms exhibit limited declarative value in salutogenetic, therapeutic contexts (Schuldt 2015). This is further exemplified by research on the neurotoxicity of amphetamine (common ADHD medication). Medication paradigms similar to therapeutic regimes evidence positive effects, whereas regimes resembling human abuse pattern reveal neurotoxic effects (Advokat 2007). In conclusion, the vast majority of research concerning “Ecstasy” appears to be inapplicable in order to establish the risks and potentials of the application of MDMA in clinical settings (Cole and Sumnall 2003; Krebs and Johansen 2012).

## What Does MDMA-Assisted Therapy Look Like?

The current treatment manual for MDMA-assisted psychotherapy proposes a mainly non-directive, patient-driven method by emphasizing empathetic presence and listening and non-directive communication. Its basic premise indicates that the interaction of the medicine, the therapeutic setting and the mindsets of participant and therapist compose the therapeutic effect. Detachment from the feeling of imminent threat while still being able to access traumatic memories may help to make engagement more comfortable and to confront the trauma without being overwhelmed. Further, an inherent feeling of safety is often noticeable, along with accelerated emotional processing. Despite appearing different from conventional treatments, MDMA-assisted therapy includes familiar elements from other models of therapy that are vital for its effectiveness: Exposure therapy, therapeutic alliance, anxiety management training, stress inoculation training, cognitive restructuring and transference and countertransference and individual therapists are encouraged to include therapeutic interventions based on their own experience, intuition, training and clinical judgment (Mithoefer 2013, 2016). The manual was created and implemented by the non-profit Multidisciplinary Association for Psychedelic Studies (MAPS) in the pursuit of approving MDMA-assisted psychotherapy as a therapeutic intervention until 2021 (Emerson et al. 2014).

Including two to three substance-assisted sessions, the treatment is usually comprised of 15 therapy sessions overall. The sessions can be subdivided into three stages: A preparatory stage (usually consisting of three 90-min sessions), followed by one substance-assisted session (including an overnight stay at the facility), succeeded by an integration stage (of several sessions). Systematic trauma exploration does not take place until the first substance-assisted session. The sessions with MDMA assistance take place 2–6 weeks apart and each one lasts around 6–8 h. Currently, 125 mg of MDMA is regarded a full dose. Typically, patients rest in a lying position while one therapist sits on each side of them. Therapist teams are composed of one male and one female therapist in order to allow for parental transference to emerge. Trauma exposure is done using a non-directive and patient-driven approach. Normally, the contents of the trauma erupt spontaneously while the therapists encourage patients to revisit and reprocess the most distressing contents. The patients may be animated to reflect on, validate and verbalize their experiences in later parts of the sessions when peak effects of MDMA lessen. Alternations between episodes of communication and episodes of introspection, during which patients may listen to a standardized set of music and have the option to wear eyeshades, are dependent on the individual process. Subsequent integrative sessions serve the purpose of supporting patients in the long-term integration process by applying insights to daily life. A detailed depiction of the treatment can be found in the MAPS treatment manual for MDMA-assisted therapy for PTSD (Mithoefer 2016).

Therapist training in MDMA-assisted psychotherapy has been recommended. In general, therapists are suggested to have strong empathic presence, to be client oriented and to have a solid background in therapy for PTSD (e.g., Cognitive Processing, PE, EMDR or psychodynamic psychotherapy). Furthermore, it is advised that the setting of the treatment should appear like a comfortable living room (for information about the importance of set and setting see Shewan et al. 2000). Nevertheless, Basic Cardiac Life Support (BCLS) is readily available and Advanced Cardiac Life Support (ACLS) can be summoned in an adequate time frame (Mithoefer 2016).

Even though the theoretical framework for substance-assisted treatment for PTSD appears to be convincing and preliminary results seem to be promising, more empirical evidence and independent replications are needed until definite conclusions may be drawn. Mithoefer (2013) outlined the differences between MDMA-assisted psychotherapy and other psychotherapy and thereby highlighted that as of yet, clinical studies assessed safety and effectiveness instead of therapeutic mechanisms. The mechanisms of actions are still of speculative nature and have to be verified by carefully designed studies. The existing studies will be critically discussed in the subsequent section.

## What Can Clinical Trials Tell Us so Far?

The first government sanctioned MDMA-assisted psychotherapy study was a randomized, double-blind, placebo-controlled trial which found preliminary evidence for the physiologically and psychologically safe application of MDMA in the psychotherapeutic context. Compared to placebo MDMA led to reduction of PTSD symptoms in chronic, treatment resistant PTSD patients. However, the sample size was too small (*n* = 6) for firm conclusions to be drawn (Bouso et al. 2008).

The first study in the United States followed a few years later. Mithoefer et al. (2011) used a randomized, double-blind, inactive placebo-controlled design and managed to include 20 participants with chronic, treatment resistant PTSD in their study. The first stage of the experiment involved participants being randomly assigned to receiving either a placebo or a full dose of MDMA (125 mg with the possibility of a supplemental dose of 62.5 mg 2 h later), during two 8-h experimental psychotherapy sessions. Both groups received preparation and integration sessions of psychotherapy. During the second stage, the participants in the placebo group were given the opportunity to take part in an open-label crossover part of the study, receiving a full dose of MDMA as well. No serious adverse effects were found to occur during the study. Further, reported scores on the Clinician-Administered PTSD Scale (CAPS) were significantly reduced subsequent to the MDMA-assisted intervention. The rate of clinical response was 83% in the active treatment group compared to 25% in the placebo group. Nevertheless, the double-blinding did not work as intended since both the researchers and the participants could successfully tell whether MDMA or a placebo had been administered. Mithoefer et al. (2013) readministered the CAPS and several additional tests to 16 of the original participants in a follow-up to their initial study, between 17 and 74 month after the completion of their ultimate MDMA-assisted psychotherapy session. While two participants relapsed, the remainder maintained significant clinical and statistical increases in symptom relief displaying the effect of MDMA-assisted psychotherapy to be persistent over time. None of the subjects associated any harm with their participation in the study.

The most recent study establishing the safety and efficacy of MDMA-assisted psychotherapy was the first one to include active placebo control into the randomized, double-blind study design (Oehen et al. 2013). In the first stage of the study the 12 participants were randomly enrolled into either an active control group receiving a low dose of MDMA (25 mg, with a supplemental dose of 12.5 mg) or the treatment group ingesting a full dose of MDMA (125 mg, plus a 62.5 mg supplemental dose). Again, the blinding was broken in the second stage of the study enabling participants to partake in a full dose MDMA-session in an open-label crossover section. Those who showed an inadequate clinical response to the full dose of MDMA had the opportunity to participate in two further sessions with higher doses (150 mg, with a 75 mg supplemental dose) in a third stage of the study. Subjects participated in either two or three substance-assisted sessions, with researchers reporting three sessions to exhibit significantly superior effects than two sessions. Although, no significant reductions in CAPS scores were found, improvements could be evidenced in the German version of the Posttraumatic-Diagnostic Scale (PDS). Results at 1-year follow up reported a 36% decrease in CAPS scores compared to their initial scores in those who received a full dose of MDMA in the first stage. The CAPS score of those who received MDMA in the second stage dropped by 52%. Since PTSD patients generally recover over time and all subjects were provided the opportunity to take part in MDMA-assisted session eventually, recovery may have been natural. The effect size for secondary outcome measures was not reported by Oehen et al. (2013). Later, others found it to be quite large (Hedges’ *g* = 0.97; Chabrol and Oehen 2013).

At present, only three therapeutic phase-two trials have been completed and published, while multiple others are currently ongoing or being prepared. An intention-to-treat (ITT) analysis of safety and primary efficacy data from six phase two trials with 105 blinded subjects shows promising results and low rates of adverse effects. Overall, a remission rate of 66.2% and an average drop of 47.7 points on the CAPS were evidenced (see Emerson 2016). Furthermore, a preliminary meta-analysis comparing the effects of PE therapy with those of MDMA-assisted psychotherapy displays promising results in favor of the latter. MDMA-assisted psychotherapy appeared to have larger effect sizes than PE therapy in clinician-observed outcomes as well as in patient self-report outcomes (Amoroso and Workman 2016).

## What is the Future Perspective of MDMA-Assisted Psychotherapy for PTSD?

Despite those phase-two trials already discussed, there are multiple others that are currently being conducted examining MDMA’s significance with regard to the treatment of MDMA-assisted Cognitive-Behavioral Conjoint Therapy (CBCT) for chronic PTSD (https://clinicaltrials.gov/ct2/show/NCT02876172), its value for social anxiety in autistic adults (Danforth et al. 2016) and anxiety associated with life-threatening illnesses (http://clinicaltrials.gov/ct2/show/NCT02427568).

Recently, the first clinical phase-three trial for MDMA-assisted psychotherapy in PTSD was approved by the FDA (Philipps 2016), allowing for a broader collection of data with more participants in order to strengthen the scientific evidence. Moreover, the FDA has grated breakthrough therapy designation for MDMA-assisted psychotherapy for PTSD (Wan 2017). So far, the Bouso et al. (2008) and the Mithoefer et al. (2011) studies were both entirely funded by MAPS. The association participated in the design and data analysis of the Mithoefer et al. (2011) study. Further MAPS partially funded the Oehen et al. (2013) study and helped to monitor and design it. This one-sided involvement in research provoked some people to question their objectivity (Sepkowitz 2012). Even though independent funding has been difficult to obtain, more research is warranted, preferable with other independent research teams involved to expand the validity and reliability of research findings.

In summary, preliminary results regarding MDMA-assisted psychotherapy for PTSD look promising and warrant further investigation. If future phase-three trials may indeed replicate these findings implication of an efficient adjunct to psychotherapy is indispensible. MDMA may provide a bridge to effectively overcome the gap between psychotherapy and psychopharmacology, thereby facilitating the integration of a more holistic approach to psychopathology (Schuldt 2015).
