# Correction to: Current Perspective on MDMA-Assisted Psychotherapy for Posttraumatic Stress Disorder

**DOI:** 10.1007/s10879-018-9382-2

**Published:** 2018-02-01

**Authors:** Sascha B. Thal, Miriam J. J. Lommen

**Affiliations:** Department of Clinical Psychology and Experimental Psychopathology, University of Groningen, Grote Kruisstraat 2/1, 9712 TS Groningen, The Netherlands

## Correction to: Journal of Contempory Psychotherapy 10.1007/s10879-017-9379-2

The original version of the article unfortunately contained a mistake in Abstract and in text under “What Does MDMA‑Assisted Therapy Look Like?” section.

In abstract, the phrase “MDMD-assisted psychotherapy” has been changed to “MDMA-assisted psychotherapy” and the correct sentence should read as below:

“Empirical support for the use of MDMA-assisted psychotherapy, including the randomized, double-blind, placebo-controlled trails that have been conducted since 2008, is discussed.”

In “What Does MDMA‑Assisted Therapy Look Like?” section, the phrase “three 90-min sessions” was incorrectly published as “39-min sessions”. The corrected sentence is given below:

The sessions can be subdivided into three stages: A preparatory stage (usually consisting of three 90-min sessions), followed by one substance-assisted session (including an overnight stay at the facility), succeeded by an integration stage (of several sessions). Systematic trauma exploration does not take place until the first substance-assisted session.

This has been corrected in the original version of the article.

